# Systems Biology Applications in Revealing Plant Defense Mechanisms in Disease Triangle

**DOI:** 10.3390/ijms26157318

**Published:** 2025-07-29

**Authors:** Tahmina Akter, Hajra Maqsood, Nicholas Castilla, Wenyuan Song, Sixue Chen

**Affiliations:** 1 Department of Biology, University of Mississippi, Oxford, MS 38677, USA; takter3@go.olemiss.edu (T.A.); hmaqsood@olemiss.edu (H.M.); ncastill@go.olemiss.edu (N.C.); 2 Department of Plant Pathology, University of Florida, Gainesville, FL 32611, USA; wsong@ufl.edu

**Keywords:** systems biology, crops, microbiome, molecular imaging, single-cell sequencing, disease triangle

## Abstract

Plant diseases resulting from pathogens and pests constitute a persistent threat to global food security. Pathogenic infections of plants are influenced by environmental factors; a concept encapsulated in the “disease triangle” model. It is important to elucidate the complex molecular mechanisms underlying the interactions among plants, their pathogens and various environmental factors in the disease triangle. This review aims to highlight recent advancements in the application of systems biology to enhance understanding of the plant disease triangle within the context of microbiome rising to become the 4th dimension. Recent progress in microbiome research utilizing model plant species has begun to illuminate the roles of specific microorganisms and the mechanisms of plant–microbial interactions. We will examine (1) microbiome-mediated functions related to plant growth and protection, (2) advancements in systems biology, (3) current -omics methodologies and new approaches, and (4) challenges and future perspectives regarding the exploitation of plant defense mechanisms via microbiomes. It is posited that systems biology approaches such as single-cell RNA sequencing and mass spectrometry-based multi-omics can decode plant defense mechanisms. Progress in this significant area of plant biology has the potential to inform rational crop engineering and breeding strategies aimed at enhancing disease resistance without compromising other pathways that affect crop yield.

## 1. Introduction

**Figure 1:**
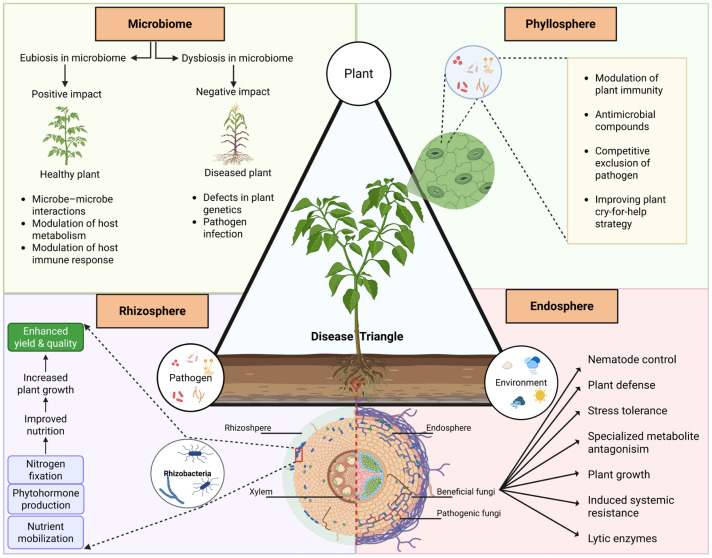
Plant disease triangle (host, pathogen, and environment) and its intricate interplay with microbiome as the fourth dimension, as well as the influences of rhizosphere, endosphere, and phyllosphere environment. This figure was created in BioRender (https://BioRender.com/rxuum3b, accessed on 24 July 2025).

Plants engage in dynamic interactions within their environments, as their survival hinges on their ability to respond to their surroundings and to attacks from microbes and pests [1]. Microbial diseases and damages, caused by viruses, bacteria, filamentous pathogens (fungi and oomycetes), nematodes, and insects, result in significant yield loss worldwide, posing severe threats to food security [2,3]. The pathogens and pests exploit plants as a nutrient-rich resource, essential for their life cycles [4]. While the genetic composition of the pathogen/pest and the plant host significantly influences the interaction’s outcome, environmental conditions often impact the incidence of disease outbreaks or infestations. The established “disease triangle” theory illustrates the significant influence of environmental factors on disease manifestation within plant–pathogen interactions; a disease outbreak requires the presence of (i) a susceptible host, (ii) a virulent pathogen, and (iii) an environment favorable to disease progression [5,6] (Figure 1). A plant may become increasingly susceptible to diseases and pests due to changes in environmental conditions [7,8]. Furthermore, plant growth and yield are significantly influenced by abiotic variables, including dynamic temperatures, altered precipitation patterns, and increased concentrations of soil salinity and atmospheric CO_2_ [9]. Field studies have demonstrated interesting interplay between abiotic and biotic factors [10,11,12]. For example, high humidity and temperatures affect the host plant’s immune response [13]. Abiotic stresses may alter the synthesis of phytohormones and other defense signals that facilitate responses to infections. Furthermore, with the advancement in rapid and cost-effective characterization techniques, a fourth dimension of host-associated microbiome has been revealed in the disease triangle that plays a significant role in plant defense and growth [6].

Recent studies highlight the critical involvement of the plant microbiome in disease resistance, even though plant disease resistance has historically been considered as a feature dictated by the pathogen virulence system and the plant innate immunity [14] (Figure 1). Interactions between plants and their microbiomes (as well as microbiomes and pathogens) are essential for various facets of host growth and development, including nutrient intake, stress resilience, and disease suppression [15,16,17,18,19]. Beneficial microorganisms may be attracted to the roots of pathogen-infected plants by emitting volatile organic compounds (VOCs) or altering the synthesis and secretion of specific root exudates [20,21,22,23]. These microbes form the rhizosphere microbiome. Similarly, those that reside internally and on the plant surface are termed the endosphere and episphere microbiomes, respectively (Figure 1). An increasing amount of evidence has demonstrated that the rhizosphere, phyllosphere, and endosphere are the crucial areas for plant growth and defense [24,25]. Protective plant rhizosphere microbiomes have been substantially responsible for the microbially mediated suppression of soil-borne diseases. It was shown that plant-beneficial microbes secrete antimicrobial compounds to deter pathogens [2]. The endosphere microbiome plays an important role once the pathogens pass the first barrier of defense and physically interact with the host cortical cells [26]. Thus, altering the plant microbiome is emerging as an environmentally friendly way to shield plants against infectious diseases and increase agricultural output. Utilizing the plant microbiome to enhance plant health and augment crop yield necessitates a foundational comprehension of the ecological patterns governing the assembly, co-occurrence, and functions of plant-associated microbiomes, as well as the ways in which plants alter their microbiomes in response to external stresses [27,28].

Owing to recent advancements in systems biology, e.g., sequencing technologies, liquid chromatography mass spectrometry (LC-MS) and CRISPR-Cas, researchers have greatly improved our understanding of plant defense systems. In particular, multiple molecular pathways and regulatory networks within the disease triangle have been elucidated [3,29,30,31,32]. The results had a significant impact on our understanding of the dynamics and complexity of interactions between plants, microbes, pathogen, and environment. Additionally, they facilitated the correlation of microbial diversity with plant traits, such as tolerance to biotic and abiotic stresses [33,34]. In this review paper, we summarize research conducted on the applications of systems biology, which consists of, but is not limited to, molecular imaging, metagenomics, and multi-omics, toward revealing the molecular interactions within the plant disease triangle [6,35]. These studies encompass the application of contemporary techniques, such as the use of high-throughput sequencing to analyze taxonomic distributions and composition of microbial communities, investigate compatible and incompatible responses, examine guard cell immunity and hormonal responses [36], employ the SynCom idea to form microbial consortiums [37], utilize MS techniques for molecular imaging of biomolecules [38], and employ a stable isotope-labeled metabolomic approach for the understanding of microbe responses [39,40]. Furthermore, functional proteins and metabolites are elucidated by proteomics and metabolomics, which investigate the ultimate products of gene expression [35,41]. Figure 1 illustrates the modified disease triangle, in which microbiomes are recognized as the fourth dimension alongside the plant, pathogen, and environment, as mentioned above [6]. It depicts the interactions between the plant and its rhizospheric, endospheric, and phyllospheric microbiomes, which facilitate growth, development, and resilience to biotic and abiotic challenges. Furthermore, this review emphasizes the use of systems biology in elucidating the intricate genetic, microbial, and metabolic networks that regulate plant defense mechanisms. Recent advances in microbiome research, multi-omics integration, molecular imaging and single-cell omics are highlighted in the review, which also indicates future research objectives and gaps. Research progress in these areas will provide important insights into plant defense mechanisms, their functions with associated microbiomes, and environmental challenges.

## 2. The Role of Microbiomes in Disease Triangle

It is well known that plants recruit and/or interact with microorganisms (bacteria, fungi, viruses, etc.), which are pivotal for their growth and development. The range of a plant’s susceptibility to diseases is based on not only its genetic makeup but also the association with beneficial microorganisms in different tissues [42]. For example, the rhizosphere acts as its first line of defense against fungal root diseases. When a pathogen breaks the first line of defense, it encounters the basal and induced defense mechanisms, which may be termed as the second line of defense. In this stage of invasion in plant roots, the endophytic microbiome may provide an additional layer of protection against pathogen attack [43]. Based on plant–microbe interaction, there are different types of interactions, e.g., beneficial (positive), mutual (positive), commensal (neutral), and pathogenic interactions (negative) [44,45]. Over the past few years, there have been studies on microbiome diversity and their relative abundance on different taxonomic groups in the rhizosphere, endosphere, or phyllosphere, yet their functional importance (e.g., activation and/or suppression against disease development) is largely unknown [42]. The rhizosphere is defined as a soil zone of 1–10 mm immediately surrounding the roots with root exudates, dead plant cells, and other entities under the plant influence. This sphere of microbiome consists of a diverse array of microorganisms, including bacteria, fungi, oomycetes, protozoa, algae, nematodes, archaea, and even viruses. Among them, the frequently studied and abundant beneficial organisms are mycorrhiza, rhizobium bacteria, and plant growth-promoting rhizobacteria (PGPR). Architectural and functional changes occur due to the balancing of basic conditions, e.g., pH, nutrient uptake, and so on, by the plant roots [46,47,48]. Balancing is required for the activation or suppression of the growth and development of specific microbial genera, thereby creating an optimal environment for plant growth, development, and fitness [49,50]. The microbial community follows a selective mode of abundance; specifically, the most abundant and functionally well-characterized microbial species are mostly found in bulk soil (which is not stuck to the root), then found in decreasing amounts in rhizosphere soil (soil that is attached to the root) and endosphere, with the lowest amount in the phyllosphere region. Endosphere microbiomes include some microorganisms (specifically known as endophytes) that penetrate the plant tissues. Unlike the rhizosphere, endophytes can grow in any part of the plant, like the root, leaf, and stem, and they live inside the tissues [42]. In contrast, those microorganisms living outside the tissues form the episphere microbiome [51,52]. The phyllosphere consists of the aerial surface of the plants, like the stem, leaves, flowers, and fruits. Unlike the rhizosphere and endosphere, the phyllosphere is considered to have less nutrient availability, more environmental fluctuations, and thus temporal variations in microbial population. It has been reported that, although there have been similarities between the microbial communities of rhizosphere and bulk soil, very small similarities are observed between the phyllosphere microbes and the surrounding open-air microbes [52]. One of the most abundant bacteria in the rhizosphere is PGPR. They are involved in several beneficial functions, including plant growth promotion, inducing tolerance to pathogenic diseases as well as abiotic stresses like drought, salinity, excess or reduced nutrient availability, and heavy metals [53].

For pathogens that overcome the first line of defense barrier, other strategies of defense mediated by microorganisms involve competitive exclusion, antibiosis, etc. [45]. There are some interesting studies; for example, a nonpathogenic strain of *Pseudomonas protegens* CS1 produces siderophore pyochelin to control citrus canker [54] and volatile organic compounds (VOCs), namely terpenoids, successfully enhanced the resistance of maize plants against pathogens [55]. However, further research is needed to understand the interactions among phytochemicals and microbial metabolism, phyllosphere non-pathogenic microorganisms, and different plant parts like the leaves, stem, and specific cells like guard cells and epidermal cells, as well as between the beneficial and pathogenic microbes. Under the pressure of pathogen attack, plants undergo changes in their metabolic pathways, resulting in chemical composition modulation of the plant’s surrounding rhizosphere. This rhizosphere modulation can result in the recruitment of protective and antagonistic microorganisms to suppress the pathogen infection [56,57]. For example, barley plants infected with *Fusarium graminearum* enriched the rhizosphere microbiome with antifungal microbes [56]. *Serratia marcescens* secretes several hydrolytic enzymes that protect tea plant from root rot disease [58]. *Flavobacterium* species from the rhizosphere soils of the Allium plants suppressed Fusarium wilt on cucumber seedlings and also inhibited the multiplication of the pathogen in soil [59]. *Bacillus* spp. is well known to secrete several metabolites not only to promote plant growth but also to inhibit pathogenic microbial growth in soil or kill pathogens through degrading the cell walls and protect rice from bacterial leaf blight [60,61]. Another interesting finding is that the host plant has an impact on their associated rhizosphere bacterial community, which can be improved by multi-cycle planting in the presence of infection [62]. For instance, the soilborne fungal pathogen AG8 was inoculated into soil during multi-cycle wheat planting. Another batch of wheat was planted without AG8 inoculation in the soil. The bacterial communities recruited to the AG8-infected rhizosphere were different from those without AG8 infection, indicating that pathogen application modifies the bacterial community composition in the rhizosphere driven by successive plantings. This enhances the development of disease-suppressive soil [63]. A similar result was found in tomato root exudates. Tomato plants were challenged with the pathogen *Ralstonia solanacearum*, and the root exudates were added to unplanted soil. The presence of pathogens in root exudate changed the exudation of phenolic compounds, increased the release of caffeic acid, and developed distinct soil microbiome communities. Caffeic acid further suppressed the growth of *R. solanacearum* [64]. A similar result has been found in ginger against bacterial wilt [65].

Recently, synthetic microbial communities (SynComs) have gained attention as a promising field of study in the biotechnology of plant defense. SynComs are small consortia of microbes of interest that are created in the laboratory based on beneficial microbiome, promoting the growth of host plants. This new technology can be a potential “green” alternative to chemical pesticides, which cause environmental contamination [37,66,67,68]. Studies revealed how plants control the rhizosphere community of bacteria by recruiting helpful microorganisms to suppress pathogenic microbes like soilborne fungal pathogens. One study showed that using cultivation-based approaches, seven SynComs derived from 14 tested bacteria from wheat rhizosphere were able to suppress *Rhizoctonia solani* AG8 infection in wheat [62]. Another study reported experiments with different combinations of bacteria and fungi as microbial consortia and separately along with different inoculation methods under different disease conditions, such as tomato plants challenged with *Fusarium oxysporum* (a root pathogen) or *Botrytis cinerea* (a shoot pathogen). The scientists aimed to determine stable and versatile biocontrol products for plant protection against a wide range of pathogenic diseases [68]. Five SynComs from five families (namely, Ophiocordycipitaceae, Trichocomaceae, Nectriaceae, Bionectriaceae, and Hypocreaceae) improved the yield and quality of a medicinal plant *Salvia miltiorrhiza* Bge. The study also mentioned that SynComs can accumulate metabolites at different phases of the biosynthesis pathways, enabling the medicinal plant to have high yield and quality by regulating the primary and secondary metabolic processes [69]. Another related finding showed that several key microbial taxa became more abundant with SynCom inoculation than control, and this increase correlated positively with pepper plant growth metrics. Specifically, the abundances of organisms like *Scedosporium*, *Sordariomycetes*, *Pseudarthrobacter*, *norank SBR1031*, and *norank A4b* rose significantly in SynCom-treated plants. These changes were linked to improved growth indicators (e.g., greater shoot height, stem diameter, biomass, chlorophyll content, leaf number, root vigor, root length, and root surface area). SynCom inoculation can effectively regulate root morphology by regulating rhizosphere microbiome and increasing key taxa abundance like *Sordariomycetes* and *Pseudarthrobacter*, thereby benefiting nutrient acquisition, resistance improvement, and pathogen resistance [70]. Although these studies have planted hopeful “seeds” towards the commercialization of biocontrol agents, there are still key knowledge gaps. For example, very little is known about how the SynComs cause metabolomic changes and/or influence both the bacterial communities and the host plants themselves. Additionally, what are the signaling and metabolic pathways impacting mutual relationships for combatting pathogenic infection and abiotic stress?

**Figure 2:**
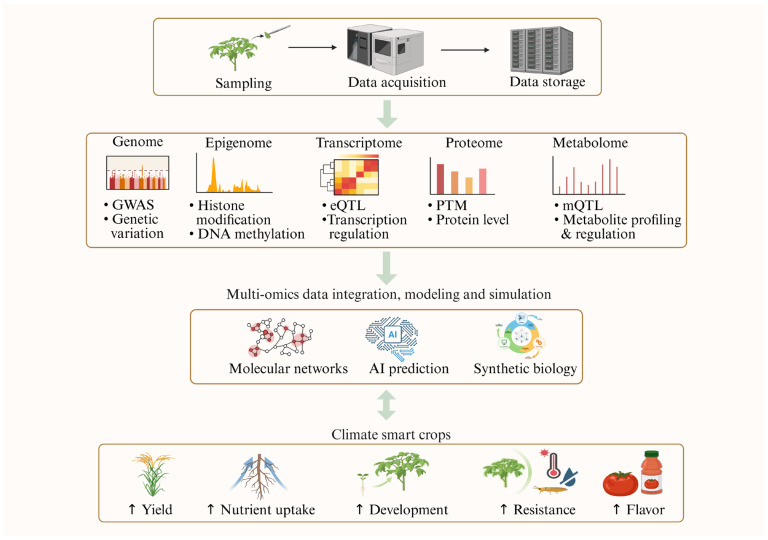
Diagram depicting systems biology workflow and its role in developing improved crops. Systems biology encompasses genomics, epigenomics, transcriptomics, proteomics, and metabolomics, as well as data integration, modeling, and simulation. The results inform AI algorithms and synthetic biology in an effort to enhance sustainable agriculture/climate smart crops, disease management, and high-quality crops. GWAS—genome-wide association studies; eQTL—expression quantitative trait locus; PTM—posttranslational modification; mQTL—metabolite quantitative trait locus; AI—artificial intelligence. This figure was created in BioRender (https://BioRender.com/kj1gx9j, accessed on 24 July 2025).

Although there is a significant amount of information on what microbes are present in different spheres and their interaction with the hosts, our mechanistic understanding of the disease triangle and utilization of systems biology remain limited. There is hardly any information on the signaling and metabolic networks underlying the plant–microbe interactions in the context of the disease triangle. Dynamic environmental conditions pose multiple simultaneous stresses to plants. For example, combined drought and heat can alter disease susceptibility. Likewise, contrasting factors such as high humidity (which opens stomata) versus elevated CO_2_ (which closes stomata) have different impacts on stomatal immunity. Finally, diverse microbes—beneficial, neutral, and pathogenic—interact in complex ways during plant disease situations. There must be metabolomic changes to both plants and microbes during the defense. The recently developed isotope-labeling of microbes may help differentiate plant metabolites from microbial metabolites [71]. There are several phytochemicals (e.g., phytohormones and phytotoxins) in different cell types involved in a plant’s defense against pathogens. Single-cell omics technologies will greatly enhance the resolution needed to better understand the plant disease triangle [72]. Advances in this area will likely enable cell-type-specific engineering so that defense may be enhanced at no compromise to yield productivity. Furthermore, very few studies have examined beneficial microbes’ modes of action during their interactions with host plants and defending plants against pathogenic microbes; such kinds of circumstances really needed to be brought to light. Therefore, the subsequent section elaborates on recent advancements in systems biology that facilitate an enhanced understanding of the plant disease triangle by unraveling the multi-layered molecular responses through integrative systems biology approaches (Figure 2).

## 3. Recent Advancement in Systems Biology of the Disease Triangle: Plant, Pathogen, and Environment Interactions

### 3.1. Molecular Imaging Toward Studying Plant–Microbe Interactions

Matrix-assisted laser desorption ionization (MALDI) MS has become one of the revolutionary technologies among the sophisticated molecular imaging techniques due to its rapid and straightforward methodology. This technology used to have a multitude of applications in analyzing biomolecules like proteins, lipids, and nucleic acids. It has become an emerging tool and a standalone method for the identification of a broad range of microorganisms, including fungi, bacteria, mycobacteria, and even viruses, because of its simple sample preparation, low cost, and time efficiency [73,74,75,76]. However, its application is still in a primitive phase regarding plant–pathogen interaction-related microbial identification. One possible reason behind this is a lack of spectral libraries of specific strains and their specific genera/species/subspecies that the identity of the unknown microorganisms can be matched to. Another factor is the level of accuracy of the result due to variations in sample preparation, type of matrix used, and the reference MS spectral libraries. Four different MALDI–Time-of-Flight (TOF) MS instruments, namely Bruker (Bremen, Germany), VITEK MS (Marcy l’Étoile, France), MicroIDSys (Suwon, South Korea) and Axima Assurance system (Kyoto, Japan) are available for MS-based microbial identification, conferred with a reference spectral database of around 5000 bacterial and fungal species. Among them, the Microflex has been the most intensively used because a maximum of 100 spectra of the unknown test spectra can be processed, from which one representative spectral profile is generated based on a defined signal/noise ratio [76,77,78]. The representative spectrum is further used for searching for similar matches in the database for determining the specific species. Whereas in case of the Axima Assurance system, similar peaks from a minimum number of strains of the same species (typically 15) are considered to build a reference super spectrum (SS) [79], in the case of Vitek MS, a single SS is generated from different test strains. That SS having a peak of above 70% among the strains is the representative of that specific species [77].

Although these MALDI biotypers have made it easier to identify microorganisms, they have less implication in the field of plant-related pathogenic microbes’ identification compared to clinical and pharmacological applications. There could be several reasons. First, it is not easy to deal with plant samples because of the presence of trichomes, the cell wall, and interfering surface chemicals, which vary under different growth conditions. Second, there is a lack of basic expertise in both the fields of plant biology and MS technologies. In this case, collaborative research between plant biologists and MS scientists can overcome this limitation; still, the interest in implementing modern technologies in plant–microbe related research needs to grow. Recently, Sivanesan et al. [74] detailed a methodology of MALDI MS for the direct identification of plant-related bacteria and fungi, where among the list of twenty, nine were related to pathogenic microbes that causes plant diseases, e.g., *Pseudomonas syringae pv. tomato* (*Pst*) causing blight as well as leaf spot in Quinoa [80], *P. grimontii* causing rot disease in Japanese turnip [81], and sheath blight and bacterial leaf blight pathogens *Rhizoctonia solani* and *Xanthomonas oryzae pv. Oryzae*. Others are endophytic bacteria, which are focused on in the study of beneficial bacteria like the plant growth promoting bacteria through nitrogen fixation, siderophore production, etc., bacteria facilitating selenium hyperaccumulators, as well as bacteria related to medicinal plants, potential biofertilizer, and many more [74,82,83,84,85].

There are still lots of plant diseases caused by microbes that need to be detected and identified and there is work to be done on understanding the impact of these microbes on different types of cells (e.g., guard cells) under different growth stages and environmental conditions. In addition, there should be more studies focusing not only on endophytic microbes but also on plant–microbe interaction and plant diseases related to the microbes in the rhizosphere and phyllosphere. Such studies will deepen our understanding of plant disease conditions and microbial communities in a broad spectrum. MALDI-TOF MS was used to distinguish plant pathogenic fungi, namely *Phytophthora infestans*, causing blight disease in tomato plants based on protein fingerprints [86,87]. There is another fascinating study on tomato metabolite identification using LC-MS and metabolic profiling using MALDI MS imaging, resulting in early detection of late blight on asymptomatic tomato plants. Tomatidine was found to be a significant biomarker of infection, with saponins as early infection biomarkers and isocoumarin as a marker of both the early and late stages of asymptomatic infection [88]. The application of the state-of-the-art molecular imaging technology in pathogenic microbe identification allows the early detection of disease (even in asymptomatic plants), informs disease management, and ultimately assists in the design of innovative strategies for enhancing plant defense and microbiome against pathogens.

### 3.2. Single-Cell Systems Biology to Revealing Plant Cell Responses to Pathogens

Unraveling novel molecular mechanisms of plant–pathogen interactions is a must to improve crop production, mitigate disease conditions, and ultimately achieve food security for humanity. For decades, scientists relied on conventional breeding to improve plant resistance against pathogens, which was time-consuming and relatively non-specific. As to the interaction between plants and microbes, the site where microbes attack on a plant is called the primary infection site, and the molecular responses are referred to as the local response [36]. There are different layers of plant defense mechanisms, namely pathogen/microbe associated molecular pattern and effector triggered immunity, as well as distal defense, i.e., systemic acquired resistance (SAR) [89].

**Figure 3:**
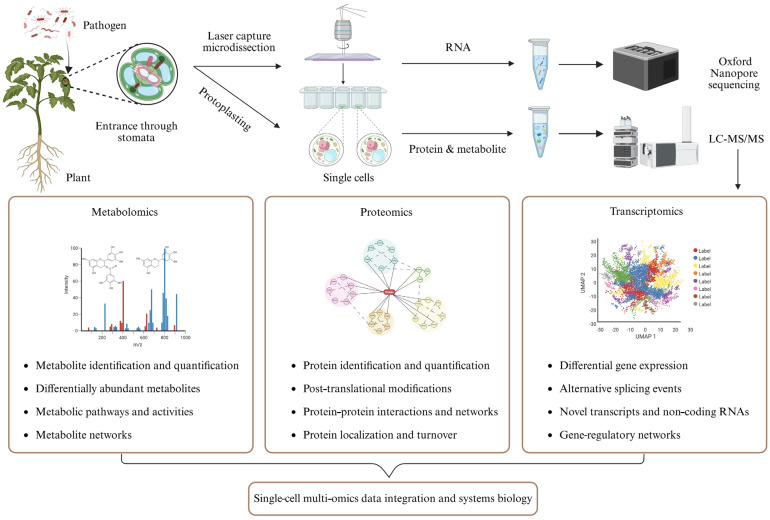
Single-cell multi-omics workflow for studying plant–pathogen interactions. After pathogen infection, plant single-cell samples are generated using either protoplasting or laser capture microdissection. The extracted RNA, protein, and metabolite samples are then subjected to sequencing and liquid chromatography tandem mass spectrometry (LC-MS/MS) data acquisition and informatic analyses. Systems biology tools are used to integrate multi-omics data toward elucidating the regulatory and metabolic networks at the single-cell level. This figure was created in BioRender (https://BioRender.com/n95s9y8, accessed on 24 July 2025).

The studies related to the molecular defense mechanisms using RNA-seq analysis often focuses on the whole tissue or organ level. A devastating plant disease called soft rot caused by *Pectobacterium atrosepticum* (*Pba*) was studied to understand the defense mechanism as well as the interaction between tobacco leaves and stems and the pathogen. To identify the transcription factors (TFs) playing a crucial role in disease progression, differentially expressed genes (DEGs) were identified based on the RNA-seq datasets. Among the DEGs, TFs WRKY6, 42, 45, 51, and 57, and TCP3 and 15 were found to be involved in plant defense against the *Pba* infection [90]. Such studies at the organ and tissue levels are useful, but they limit our understanding of the intricate molecular regulations at the cellular level. Nowadays, single-cell RNA sequencing has become more and more widely used, including studying plant responses to pathogens [91,92,93,94] (Figure 3). Through protoplasting, single cells were isolated from *Arabidopsis thaliana* leaves after infection by a fungal pathogen *Colletotrichum higginsianum*. The results revealed cell-type-specific DEGs, particularly thee enrichment of intracellular immune receptors in the vascular cells [95]. Another study employed the single-cell RNA sequencing of over 50,000 forest strawberry cells to elucidate the DEGs during a necrotrophic fungus (*Botrytis cinerea*) infection. Predominant cell types, unique gene expression profiles, and elevated expression of disease resistance-related genes and TF-encoding genes were identified [96]. Plant responses to the pathogens are heterogeneous in nature. For example, there is a significant difference between immune and susceptible cell cluster marker expressions. The expression patterns of immune cluster markers are spatial and temporal, whereas susceptible marker expression patterns are expansive and sustained in response to *Pst* DC3000 infection in *A. thaliana* [97]. Although uniform bacteria/fungus inoculation to the plant tissue is ensured, pathogens penetrate and colonize the leaf tissue unevenly, which creates variation in response by different plant cells. During protoplast generation, transcriptional changes may occur that result in the failure of detect some potential defense related genes. Also, over the years, single-cell transcriptomic profiling has focused on defense-related genes, lagging behind are the susceptible genes and the microbe-related gene expression profiling, which can be potential targets for developing disease-resistant crops.

Compared to single-cell transcriptomics, single-cell proteomics and metabolomics are in their rudimentary stage and substantially more challenging. A few limiting factors include the discrepancy between detected proteins and total number of genes, a wide range of post-translational modifications [98], and only about 14,000 metabolites out of potentially a million in the plant kingdom having been identified [99]. To date, there are some studies on single-cell proteomics of tomato roots by laser capture microdissection (LCM) followed by gel- and LC-MS/MS-based proteomics analysis for the identification of structural and functional proteomes contained in individual cell layers [91]. Recently, Montes et al., conducted protoplasting to separate two adjacent Arabidopsis root cell-types and identified 1118 proteins per cell from a total of 756 cells. The relative levels of the proteins were quantified in individual plant cells [92]. Metabolite profiling in a single cell type has also been performed in Arabidopsis roots using a workflow for metabolomic analysis of single cell type populations. Fifty metabolites were putatively identified, with the most prominent groups being glucosinolates, phenylpropanoids, and dipeptides [93]. The use of single-cell multi-omics in plant disease triangle studies has yet to be reported. A conceptual framework in is given Figure 3 to shed light on the future of single-cell multi-omics integration and systems biology. Although this is challenging, if we can work on identifying potential proteins and metabolites that play a role in defense against or susceptibility to pathogen attack, they can be potential targets for cell-specific genetic engineering for crop enhancement against diseases.

### 3.3. Metagenomics, SynComs, and Multi-Omics Integration/Systems Biology

Metagenomics has been widely used in the genetic analysis of genomes followed by functional expression analysis and random shotgun sequencing of environmental DNA. Metagenomics plays a critical role in uncovering enormous functional gene diversity in the microbial community to know more about the phylogenetic order and functional and structural information of the microbial community, as well as the ecological and evolutionary profiles of microbes and microbial communities [41]. As a modern technique, metagenomics besets more genetic information than traditional approaches that unlock many biotechnological potentials in crop improvement and sustainable food production [94]. There are two main approaches to metagenomics, namely functional metagenomics and sequence-based metagenomics [100]. Soil is known to be the most complex and diversified terrestrial environment, where metagenomics can act as a potential technique to unlock the diverse metabolic, proteomic, genomic, and phylogenetic resources to address and shape the microbial activity, especially in the rhizosphere, otherwise stated as rhizosphere engineering [101]. It has been reported that rhizosphere microbes have some level of plant dependency; for example, they rely on plant-derived compounds for their metabolism. To represent the functional repertoire of a wild blueberry (*Vaccinium angustifolium*)-associated soil microbiome and to determine the trade-off between the rhizosphere and bulk soil metabolic capabilities of microbes, a co-occurrence analysis was performed using shotgun metagenomic sequencing [102]. This study found that the basic metabolic functions necessary for both bulk and rhizosphere microbiomes are complemented with rhizosphere-specific microbiomes. A rhizosphere-specific metabolic pathway was found to be involved in xenobiotic and terpenoid biodegradation that could enable the microbiome to functionally respond to stressed conditions [103]. This kind of study can be performed in the future by focusing on specific stress or combined stress conditions to better understand the underlying pathways to respond to or defend against the stresses. The metagenomic approach has also been utilized in the soil biochemistry field, and *nifH* genes with nitrogen fixing ability have been identified in rhizosphere soil [94]. The identification of novel bioactive components, namely halotolerant enzymes (e.g., lipase) from the soil microbes, has the potential to enable the cultivation of plants with high salinity tolerance [104]. The identification of a symbiotic relationship between microbes, for example, interaction between *Burkholderia* (a bacteria species) and the fungus *Rhizopus*, in the symbiotic relationship, spore production, and survival of the fungus that is dependent on the metabolites produced by the bacteria [105] and many more applications of metagenomics have been marked to date. Also, metagenomic profiling has been useful in studying community diversity, relative abundance of various taxa, and predicting the gene function of soil microbiota, as well as revealing succinoglycan riclin as a successful polysaccharide-type biocontrol agent to improve soil suppressiveness against Fusarium wilt by reshaping microbiota and accumulating plant-beneficial microbes [106]. Moreover, advancement in metagenomics is helping to shed light on factors affecting the successful establishment of the SynComs and their interaction with the plants, e.g., seasonal change, genotype, prevalence (consistency among plant developmental stages), and colonization efficiency, as well as their metabolic capabilities [107]. Currently, genomic information and gene expression profiles have been used to identify the functional traits of relevant microbes and design for an effective SynCom, which is less laborious than the traditional procedures like plating [102,108]. Additionally, combining machine learning, AI algorithms, and bioinformatics for the integration of different omics data from various combinations of microbial communities along with phenotypic expression datasets through different environmental parameters, plant genotypes, and stress conditions will be a critical step towards creating a benchmark of efficient SynCom.

Systems biology has been of growing interest in the exploration of plant defense mechanisms against various biotic and abiotic stresses. However, the disease triangle concept is a relatively new area to explore where biotic stresses interact with abiotic stresses. Table 1 includes a list of combined abiotic–biotic stresses related proteomic, metabolomic and transcriptomic studies in plants [109,110,111,112,113,114,115,116,117,118,119,120,121,122,123,124,125,126,127,128,129,130,131,132,133] (e.g., drought and bacterial disease in Arabidopsis [109], drought and nematode in cowpea [112], ozone and pest attack on black mustard [116], salinity and pathogen stress in tomato [132]). Although there have been significant studies on plant metabolomics [134,135,136,137,138], plant metabolites produced in the presence of plant-associated microbiomes in combination with abiotic stresses (i.e., disease triangle) are still poorly studied. There might be several reasons behind this non-development in this field, which can be the complex nature of soil where the microbiome exists, the dynamics of soil properties and plant root-associated microbiomes, and the interaction between the soil-borne microorganism and the host plant [44,139]. LC-MS and proton nuclear magnetic resonance spectroscopy (^1^H-NMR) have been utilized to study the metabolic profile of suppressive and non-suppressive soil against the soil borne pathogen *Rhizoctonia solani* AG8, which causes rhizoctonia root rot and bare patch diseases in cereal crops. The study revealed that suppressive soil is abundant in sugar molecules, which are abundant in lipids and terpenes. Also, MS fragmentation showed an abundance of “macrocarpal” in suppressive soil, which is an antimicrobial specialized metabolite [140]. Similarly to single-cell metabolomics, general metabolomics of plant–microbe interaction faces challenges. For example, there is a lack of suitable MS^n^ fragmentation spectral libraries for untargeted plant metabolomics. Also, there is a lack of spectral libraries related to soil- and rhizosphere-related microbial metabolites that provide in-depth coverage. This type of environmental metabolomics is still dependent on authentic standards, which are often not readily available. To date, it is even difficult to identify and/or quantify potential metabolites produced by the microbes associated with soil or plants, let alone in mixed microbial populations.

Multi-omics data integration allows the formation of an understanding of biological systems at different levels of genetic information flow (Figure 2). One study conducted dual transcriptomic and metabolomic analyses of flax plant against a biotrophic pathogen (*Odium lini*). The plant exhibited genotype-specific resistance to the pathogen by a rapid response marked by up-regulation of defense genes, whose protein products reside within the cell wall, and accumulation of specialized metabolites [141]. Additionally, the alterations in metabolites and gene expression in *Mikania micrantha* following infection by *Puccinia spegazzinii* were also reported using genomics and transcriptomics analysis [142]. In terms of abiotic stress, the molecular responses of *Brassica napus* to salt stress were investigated using transcriptomics, proteomics, and metabolomics. Functional enrichment analysis of DEGs, differential metabolites (DMs), and differentially expressed proteins (DEPs) identified the key players in *B. napus* response to salt stress [143]. Similar studies were conducted in other plants under other conditions, employing transcriptomics, proteomics, and/or metabolomics [144,145,146]. These studies demonstrate the significance of multi-omics integration, which has enhanced our understanding of complex pathways, regulations, and molecular networks, as depicted in Figure 2. However, in our survey of the literature on omics applications in the plant disease triangle (Table 1), multi-omics integrative studies have been rare, to date.

There are many studies conducted on stress responses of perennial plants using multi-omics approaches. For instance, transcriptome, sRNAome, and degradome sequencing was utilized to investigate the dynamic resistance mechanisms of tea plants infected with gray blight caused by Pestalotiopsis-like species [147]. A proteomic analysis was conducted on *Citrus sinensis* to elucidate the biological processes underlying the occurrence of Huanglongbing (HLB) disease caused by phloem-restricted bacterium *Candidatus Liberibacter* [148]. Similar studies focusing on transcriptomics and metabolomics were also reported [149,150]. However, these separate omics data sets were not integrated into a systemic regulatory network. Another study investigated the molecular mechanisms underlying cherry response to drought using transcriptomics and metabolomics of drought-tolerant cherry rootstock and drought-susceptible cherry rootstock. Important drought-responsive genes and metabolites were identified to be involved in cyanoamino acid metabolism and phenylpropanoid biosynthesis, and they are potential biological indicators for cherry drought response [151]. Clearly, these studies lack either a biotic component or an abiotic component to be relevant to the interesting area of disease triangle (Table 1). This current status highlights an important future direction of plant disease triangle research, i.e., incorporating the new tools and development in multi-omics and systems biology (Figure 2).

## 4. Isotope Labeling Technique: A Powerful Tool for Distinguishing Plant and Microbial Metabolites

Metabolites, especially primary compounds, are commonly found, regardless of species (e.g., plants, microbes, animals, etc.). They can also be interchanged between host and pathogen. There are a number of studies in the omics field of plant pathology; however, hardly any experiment is found where they differentiate the pathogen metabolites from the plant metabolites. This might be a minor problem for transcriptomics and proteomics because they possess established species-specific databases [152]. However, for metabolomics, we often see cross-contamination in the database. For example, even in plant metabolite databases, we see non-plant metabolites. Additionally, the broad range of shared metabolite groups adds an extra layer of complexity to quantifying metabolomic changes in host–pathogen interaction. This makes the metabolomics study challenging because to draw a conclusion of the result, it needs to match the result with the available database manually one by one to check if they are plant or non-plant metabolites. This process is very time-consuming and error-prone. To address this problem, stable isotope labeling of microbes can be a great option. Stable isotope labeling can be applied for identification and absolute quantification of metabolites in a given metabolic pathway [153,154], differentiating plant and non-plant metabolites [71], identification of metabolites in different microorganisms [39], cellular function of plant tissue metabolism under abiotic stress [155], and metabolic flux analysis [156,157]. Another powerful method is stable isotope labeling by amino acids in cell culture (SILAC), revealing the proteome of bacterial cells [158]. An interesting study has shown the effect of separating bacteria from Arabidopsis epidermal samples through stable isotope labeling of *Pst* DC3000 metabolome with ^13^C and ^15^N [71]. To remove extra bacterial cells from the Arabidopsis epidermal peels, washing with 0.85% NaCl was conducted on the *Pst* DC3000-incubated epidermal peels, which showed 95% removal of bacterial cells from the peels [71,159]. After multiple reaction monitoring (MRM) LC-MS/MS, the result showed that selected metabolites can be identified from the isotope-labeled samples, with different labeling efficiency ranging from a few to 100%. The result also highlights the role of primary metabolites (specifically, ATP and amino acids) and carbohydrates (namely glucose, fructose, and sucrose) in defense against pathogenic bacteria [71].

Ćeranić et al. presented a method to grow stable isotope-labeled durum wheat in a controlled growth chamber with hydroponic nutrient supply. Even ^15^N labeling can be achieved in standard greenhouse conditions without specialized atmospheric control [160]. The method ensures uniform isotopic labeling and can be adapted for other plant species with appropriate adjustments for environmental and nutritional requirements. These isotopically labeled plant extracts successfully resulted in 652 truly wheat-derived metabolites after isotope-assisted LC-MS analysis [160]. This kind of study can be an alternative option for plant proteomics and metabolomics study of the disease triangle. For instance, the guard cells extract of an isotope-labeled plant treated with drought, salinity, or other abiotic stress can help us find specific plant proteins and metabolites. Then, incubating the plant cells with pathogen might have less chance of cross-contamination with plant proteins and metabolites. Moreover, this kind of study will also be beneficial to conducting the plant disease triangle proteome and metabolome response and/or defense in real environmental conditions. These are unexplored areas of stable isotope labeling applications. Furthermore, the isotope-labeled metabolomics and proteomics data have not been acquired using the up-to-date LC-MS/MS techniques like sequential window analysis of all theoretical mass spectra (SWATH), MRM-HR, etc. This approach may increase the number of identified molecules with high accuracy. It should also be noted that the cost, decreased throughput, and technical complexity associated with isotope labeling should be considered when designing the experiments.

## 5. Concluding Remarks and Future Perspectives

Current climate problems have had a tremendous impact on global food security. Systems biology offers promising solutions to the challenges in plant disease triangle. There is much room for improvement in this area, especially multi-omics study to discover potential proteins and metabolites in response to the abiotic and biotic stresses, and then to engineer the molecular markers into the plant and/or the microbial community to improve plant immunity and productivity. So, enhancing research into plant defense systems under disease triangle conditions by applying multi-omics techniques is critical. In particular, the use of metabolomics together with reverse genetics is of growing interest, which is useful for identifying new metabolites along with the respective genes that will open up a new possibility of genetic engineering to develop disease-resistant plants. Novel technologies are emerging in plant multi-omics, and they offer increased sensitivity and high resolution, along with deep coverage in a high-throughput manner. For example, improved mass-spectrometric technologies like SWATH and MRM-HR will help identify more metabolites and proteins. Also, improvement in spectral libraries will continue to enhance MS^n^ spectra interpretation. There are some studies exploring the potential applications of the biocontrol agent SynCom, but this kind of study mostly focused on biotic stress, hardly addressing abiotic stress, let alone the disease triangle conditions. Moreover, single-cell resolution (for example, guard cell, mesophyll cell, epidermal cell, etc.) related to the disease triangle aspect is still a new area to explore where we can know more about what’s happening in the specific cell during pathogen infection and defense. It is high time we considered combining multi-omics with single-cell or single-cell-type materials, integrating the data across the central dogma, and revealing specific molecular mechanisms and networks (Figure 2 and Figure 3). Together with the fast development of artificial intelligence, this is where systems biology is reshaping plant pathology. A better understanding of the molecular mechanisms underlying the plant disease triangle and their applications to the agricultural field is critical for limiting crop diseases, enhancing food production, and ensuring sustainable agriculture.

## Figures and Tables

**Table 1:** Combined abiotic–biotic stresses related proteomic, metabolomic, and transcriptomic changes in plants.

Proteomics				
Host Plant	Abiotic	Biotic	Molecular Changes	Ref.
Arabidopsis (*A. thaliana*)	Drought (moderate)	*Pseudomonas* *syringae* pv. tomato	Drought suppressed SA-mediated defense by repressing CBP60g and SARD1, which decreased PR proteins. ABA levels increased under drought	[109]
Arabidopsis (*A. thaliana*)	Heat stress (with drought)	*Turnip mosaic* *virus*(*TuMV*)	Heat stress suppressed R-gene-mediated defense, allowing increased virus replication. Heat shock proteins and chaperones were highly induced, prioritizing abiotic stress tolerance over antiviral defense	[110]
Chickpea (*Cicer arietinum*)	Drought	*Fusarium* *oxysporum* sp.	Combined stress led to higher expression of PR proteins (chitinases, β-1,3-glucanases), antioxidant proteins, and osmoprotectants	[111]
Cowpea (*Vigna unguiculata*)	Drought	*Meloidogyne* spp.	Upregulation of disease-resistance proteins (NBS-LRR class), pathogenesis-related (PR) proteins (e.g., chitinase, PR-1, thaumatin), and antioxidant enzymes	[112]
Rice (*Oryza sativa*)	Drought	*Xanthomonas oryzae*	Decrease in photosynthesis and carbon metabolism proteins. Increase in receptor-like kinases, MAP kinases, ribosomal proteins, and stress-responsive translational regulators	[113]
Wheat (*Triticum aestivum*)	Drought	*Sitobion avenae*/*Metopolophium* *dirhodum*	Photosynthesis proteins were repressed under combined stress. Increased expression of mitochondrial respiratory enzymes and ATP synthase subunits, contributing to stress tolerance	[114]
**Metabolomics**				
Arabidopsis (*A. thaliana*)	Light, humidity, drought, heat and cold	*P. syringae*/*Botrytis cinerea*	Sustained metabolome changes in osmoprotectants and antioxidants (fumaric acid, flavonoids and anthocyanins) configured in response to abiotic stresses can act as modulators of plant immune responses	[115]
Black mustard (*Brassica nigra*)	Ozone (O_3_) pollution	*Pieris* *brassicae*	Ozone inhibited photosynthesis; herbivory increased volatiles and defense metabolites	[116]
Cucumber (*Cucumis sativus*)	Salinity	*P. syringae pv. lachrymans*	Salt and pathogen caused redox imbalance, ABA increase, SA suppression	[117]
Maize (*Z. mays*)	Drought	*Aspergillus* (aflatoxin)	Accumulation of simple sugars and polyunsaturated fatty acids; increased ROS	[118]
Maize (*Z. mays*)	Heat stress	*Cochliobolus* *heterostrophus*	Elevated hydroxycinnamic and p-coumaric acid levels, increasing heat-induced susceptibility	[119]
Milkweed (*Asclepias fascicularis*)	Drought	*Danaus* *plexippus*	Herbivory suppressed drought-induced flavanol glycosides; reduced defense metabolites	[120]
Potato (*Solanum tuberosum*)	Heat stress	*Phthorimaea* *operculella*	Heat suppressed herbivory-induced defensive metabolites (jasmonates, glycoalkaloids)	[121]
Rice (*O. sativa*)	Salinity	*Sitobion avenae*	Salt stress altered aphid metabolism, reducing sugar and fatty acid accumulation in aphids	[122]
Tomato (*S. lycopersicum*)	Severe drought	Tomato yellow leaf curl virus	Higher proline content	[123]
Tomato (*S. lycopersicum*)	Moderate drought	*Tetranychus* *evansi*	Drought and herbivory increased ABA and SA, respectively; mite altered osmolytes and defense metabolites	[124]
**Transcriptomics**				
Arabidopsis (*A. thaliana*)	Drought	*Pseudomona* *syringae*	Combined stress differentially regulated drought and pathogen responsive genes, including *AtNCED3*, *AtPR5*, and *AtNAC6*	[125]
Arabidopsis (*A. thaliana*)	Drought	*Heterodera schachtii*	Over 50 genes were uniquely regulated under dual stress conditions, including *AtRALFL8*	[126]
Arabidopsis (*A. thaliana*)	Heat (38 °C) and Drought	*TuMV*	Triple stresses altered 61% of gene expressions non-additively, suppressing anti-ethylene TF *Rap2.9* (*DEAR5*) under heat and drought	[110]
*Boechera stricta*	Drought	*Spodoptera* *exigua*	290 genes were upregulated under drought-herbivory stress, and *MYB13* was suppressed	[127]
Norway Spruce (*Picea abies*)	Drought (mild vs. severe)	*Endoconidiophora polonica*	Mild drought pre-stress increased resistance, severe drought suppressed defense genes	[128]
Rice (*O. sativa*)	Drought (intermittent)	*Magnaporthe* *oryzae*	Drought pre-stress dampened defense-related transcripts, increasing pathogen virulence	[129]
Rice (*O. sativa*)	Heat and Cold	*X. oryzae*	*bHLH* gene *Os04g0301500* acts as a key regulator in coordinating heat/cold and bacterial responses	[130]
Sunflower (*Helianthus annuus*)	Drought	Mixed fungal pathogens	Shared oxidative stress genes upregulated under combined stress, overlapping with defense pathways	[131]
Tomato (*S. lycopersicum*)	Salinity	*Oidium* *neolycopersici*	Stress responses varied based on salt levels; hormonal signaling (ABA, JA/ET) played a role in stress adaptation	[132]
Wheat (*T. aestivum*)	Drought	*F. pseudo-* *graminearum*	Gene co-expression analysis showed shared and distinct stress-responsive pathways; candidate genes on chromosome 2D identified (including *TraesCS2D03G1055700*)	[133]

## Data Availability

Not applicable.
